# Therapeutic effects of mesenchymal stromal cell secretome in liver fibrosis with acute lung injury

**DOI:** 10.3389/ebm.2025.10782

**Published:** 2025-09-30

**Authors:** Ane Caroline Ribeiro Novaes Martins, Karina Ribeiro Silva, Anna Carolina de Souza Pereira, Gustavo Claudino Paris, Ana Lúcia Rosa Nascimento, Verônica Aiceles, Erika Afonso Costa Cortez, Alessandra Alves Thole, Simone Nunes de Carvalho

**Affiliations:** ^1^ Stem Cell Research Laboratory, Histology and Embryology Department, Rio de Janeiro State University (UERJ), Rio de Janeiro, Brazil; ^2^ Post-Graduation Program in Clinical and Experimental Pathophysiology (FISCLINEX), Rio de Janeiro State University (UERJ), Rio de Janeiro, Brazil; ^3^ Ultrastructure and Tissue Biology Laboratory, Histology and Embryology Department, Rio de Janeiro State University (UERJ), Rio de Janeiro, Brazil

**Keywords:** liver fibrosis, acute lung injury, mesenchymal stromal cells, secretome, regenerative therapy

## Abstract

Chronic liver disease (CLD) is a widespread condition and liver fibrosis is a common hallmark. The COVID-19 pandemic has drawn awareness over emerging pathogens that pose severe risks for chronic disease patients, whose management is complicated because most drugs can overload liver metabolism, therefore therapeutic alternatives are needed. Aims: based on the difficulty of treating CLD patients during respiratory infections, this study focused on the therapeutic evaluation of adipose-derived mesenchymal stromal cell (ASC) secretome. Methods: the effects of ASC secretome were evaluated in a preclinical murine model of liver fibrosis induced by thioacetamide (TAA) and acute lung injury induced by lipopolysaccharide, using histological and cytokine profile analyses. ASC secretome exhibited therapeutic effects alleviating fibrogenesis and inflammation, decreasing plasmatic inflammatory markers (cytokines IL-6, IL-17A and TNF-α), and restoring immune homeostasis. The secretome reduced liver collagen accumulation and IL-6 levels and restored lung cytoarchitecture, decreasing levels of CD68 and TNF-α. These results provide a preclinical basis for potential clinical use of the ASC secretome and its products, advancing the concept of cell-free, systemically active interventions for complex tissue injuries, and reinforcing the potential of its paracrine factors to modify pathological responses and promote tissue regeneration in combined chronic-acute diseases.

## Impact statement

This study demonstrates the therapeutic efficacy of murine adipose-derived mesenchymal stromal cell (ASC) secretome in a dual-injury preclinical model combining liver fibrosis and acute lung injury. By showing histological and inflammatory improvements in both organs after systemic administration of minimally processed, cell-free preparation in a murine model, this work suggests the translational value of ASC-derived secretome in complex conditions offering a scalable strategy for regenerative interventions in translational pipelines, especially for real-world health systems seeking affordable, cell-free alternatives for chronic and acute inflammatory conditions. The findings contribute to new *in vivo* data confirming the systemic reach and multi-organ impact of stromal cell-derived soluble factors in comorbid diseases, providing a relevant foundation for future translational studies.

## Introduction

Chronic liver disease (CLD) prevalence has been increasing in the later years, in a widespread pattern driven by metabolic diseases associated with industrialized food intake, as well as the consumption of pharmaceutical and recreational drugs and infection by hepatitis viruses [1]. The COVID-19 pandemic impacted mainly patients suffering with chronic diseases such as liver fibrosis or its more severe condition, cirrhosis, associated or not with the metabolic changes related to non-alcoholic fatty liver disease (NAFLD) and nonalcoholic steatohepatitis (NASH) [2–4]. This group had higher morbidity and mortality rates during the COVID-19 pandemic, in a predictable scenario, since acute lung infections by different pathogens of bacterial, fungal, or viral origins are one of the main causes of death in chronic patients [4–8]. Alternatively, respiratory infections may cause liver damage as a direct impact of the pathogenic agent and/or as a secondary effect of acute lung injury (ALI), as observed for the COVID-19 pathogen, the severe acute respiratory syndrome coronavirus 2 (SARS-CoV-2) [9–11]. This demonstrates that ALI is a major concern for chronic liver disease patients, and the clinical management of these patients when both diseases happen to coexist is complicated, because many medications such as antibiotics and anti-inflammatory drugs directly affect the impaired liver. Other situations like post-transplanted patients, the occurrence of ascites and paracentesis procedures also pose greater risk of nosocomial respiratory infections that are a major mortality factor in this group [12–17].

Mesenchymal stromal cells (MSCs) are found in diverse tissues, where they differentiate from mesenchymal stem cells. MSCs stand out for their therapeutic properties, including their ability to differentiate into distinct cell types, undergo targeted migration in response to injuries, modulate the immune system, and release tissue repair factors in a paracrine way via their well-known secretome [18–20]. They were originally characterized in bone marrow and can be found in various adult tissues especially in perivascular niches, with adipose tissue emerging as a prominent source due to its availability and minimally invasive collection capabilities. Adipose Stromal/Stem Cells (ASC) are a heterogenic cell population that comprehends mesenchymal stem cells and their progeny, isolated after enzymatic digestion of the unilocular (or white) adipose tissue obtained, for instance, from lipoaspirates [21].

Therapy using MSC secretome has shown benefits in treating conditions such as hepatic fibrosis [22–26] and ALI [27–31], but there is no data regarding the effectiveness of this approach in the combined diseases, which is increasingly a likely scenario. Therefore, our study aimed to investigate the potential therapeutic effects of ASC secretome in a murine model of concomitant hepatic fibrosis and ALI, exploring its impacts in histological features and cytokine profile in both systemic (plasma) and local (lung and liver) analyses.

## Materials and methods

All animal experiments were conducted in compliance with international standards for the care and use of laboratory animals, following the ARRIVE guidelines and equivalent international recommendations, under approval of the institutional Ethics Committee (protocol IBRAG 031/2023).

### Isolation of ASCs from mouse adipose tissue

ASC were obtained from the inguinal adipose tissue of healthy male C57BL/6 mice (n = 4), 8 weeks old, which were euthanized according to the protocol approved by the Ethics Committee. Inguinal subcutaneous adipose tissue was collected and kept in a solution containing antimicrobials penicillin 500IU/mL, streptomycin 0.5 mg/mL, gentamicin 0.25 mg/mL and amphotericin B 0.012 mg/mL in DMEM (Dulbecco’s Modified Eagle Medium, Sigma-Aldrich) high-glucose at 4 °C for 2 h, and then dissociated with 0.2% type II collagenase solution (Sigma-Aldrich) in DMEM for 15 min at 4 °C followed by agitation for 45 min at 37 °C in an orbital shaker. Then DMEM with 10% Fetal Bovine Serum (FBS) (Gibco) was added to stop enzymatic dissociation. The suspension was filtered in a 100 μm cell strainer (BD Biosciences) and centrifuged at 300 *g* for 5 min. The cell pellet was resuspended in DMEM-F12 with 15% FBS and antimicrobials.

The cells were plated in 25 cm^2^ culture flasks at the rate of 1 bottle per animal used for cell isolation and maintained at 37 °C in a humidified atmosphere with 5% CO_2_. After 48 h, non-adherent cells were removed by washing the cultures with buffered saline solution (PBS, phosphate buffer saline, pH 7.4) and adherent cells were maintained in complete culture medium. When the cells reached 70%–80% confluence, the passage or replating procedure was performed using Trypsin (Gibco), incubated for 5 min at 37 °C. After 7 days cells were counted by exclusion of trypan blue dye (0.4% in PBS) in a Neubauer chamber and replated in complete medium until growth and new confluence. The procedure was repeated until the third passage, when they were plated to obtain the secretome.

ASCs in the third passage were characterized using anti-mouse CD45-FITC, CD90-APC and CD105-PE primary antibodies (BD Biosciences) and analyzed in an Accuri C6 flow cytometer. To isolate the secretome, ASCs were plated in 24-well plates, at a concentration of 7.5 × 10^4^ cells per well, in DMEM-F12 containing 15% FBS. The following day, cultures were maintained with 0.6 mL/well of DMEM-F12 without serum, to condition the supernatant with the ASC secretome. The supernatant was filtered through a 0.22 µm filter, aliquoted and stored at −80 °C for further analysis. Cell numbers at plating were adjusted to total medium volume proportions in order to maintain the same concentrations in secretome samples used in the study.

### ASC secretome characterization with NMR-based metabolomics

Nuclear Magnetic Resonance (NMR)-based metabolomics were done by Metabogen Diagnostico^®^ protocol.^1^ Several metabolites could be assigned, as shown in Figure 2. In addition, by 2D NMR spectra we could assign 4-Hydroxybenyl alcohol, 4-hydroxyphenylacetonitrile, agmatine, alanine, cadaverine, D-glucose, D-glucuronate, D-xylose, ethanol, glycine, homoarginine, l-arginine, l-canavanine, l-glutamine, l-isoleucine, l-tyrosine, l-valine, lactic acid, leucine, malonic acid, maltose, pyruvic acid, rhamnose.

### Experimental groups and induction of liver fibrosis and acute lung injury

In this work, 8-week-old male C57BL/6 mice were distributed into 3 experimental groups with n = 8 each. Except for the control group, all animals underwent peritoneal injection of thioacetamide (TAA) (Sigma-Aldrich) and nasal instillations of a single dose of lipopolysaccharide (LPS) to establish a model of concomitant chronic liver disease and ALI. To induce liver fibrosis, TAA (Sigma-Aldrich) was diluted in 0.6% sterile PBS and applied at a concentration of 100 mg/kg intraperitoneally, 3 times a week, for 6 weeks, in male C57BL mice, at 8 weeks of age [32]. The animals were transferred to cages with food and water *ad libitum* and monitored throughout the procedure. Histological analysis with Picrosirius (Sirius Red) confirmed that liver fibrosis was established after 6 weeks of TAA administration. For the induction of ALI, mice at the end of the sixth week of liver fibrosis induction (42nd day) received a single nasal instillation containing 30 μL of LPS solution in warm sterile PBS (LPS at 10 mg/mL, obtained from *Escherichia coli* O111:B4, 076K4020, Sigma-Aldrich) [33].

After 6 weeks of treatment with TAA followed by the administration of an intranasal dose of LPS, the secretome obtained from ASCs was administered in 2 doses of 0.4 mL each in the peritoneal region of the animals. Therefore, the groups of animals that made up this study were: (a) control group (CTRL), healthy animals that were not subjected to the procedures; (b) Liver fibrosis and ALI group (TAA+LPS), animals that received TAA for 6 weeks, 3 times a week, and intranasal LPS at the beginning of the sixth week (42nd day), being euthanized at the end of the seventh week (49 days); and (c) Liver fibrosis and ALI treated with ASCs secretome (namely ASC group), animals that received TAA for 6 weeks, 3 times a week, and intranasal LPS at the end of the sixth week (42nd day). After 48h, they received the first dose of 0.4 mL of ASC secretome (44th day) and the same dose on the 46th day, being euthanized on the 49th day.

On the day of euthanasia, blood samples were collected by cardiac puncture in syringes containing sodium heparin (anticoagulant, Cristalia), and transferred to an Eppendorf tube. Then, the blood was centrifuged at 2.000 rpm for 10 min at room temperature to obtain plasma. The resulting plasma was then collected and stored at −80 °C for further analysis. Liver and lung samples were harvested, fixed with 4% buffered formaldehyde, dehydrated in ethanol, clarified in xylol and embedded in paraffin for histological analysis.

### Cytokine profile analysis of the ASCs secretome, plasma, liver and lung

The presence and quantity of the cytokines IL-10, IL-17A, TNF-α, IFN-γ, IL-6, IL-2 and IL-4 were evaluated in the ASCs secretome and in plasma, livers and lungs from experimental groups using the CBA (Cytometric Bead Array) Mouse Th1/Th2/Th17 Kit (Catalog number 560485, BD Biosciences), according to the protocol recommended by the manufacturer. Liver and lung lysates protein contents were previously measured with BCA (bicinchoninic acid) protocol (Pierce, Thermo Scientific) for normalization. Samples were acquired on a flow cytometer (BD C6 Accuri) and analyzed using FCAP Array 3.0 software (BD Biosciences).

### Liver analysis

The levels of liver enzyme Aspartate Aminotransferase (AST) (also called TGO, for glutamic-oxaloacetic transaminase) were measured in plasma using commercial kits following the manufacturer’s protocol (Bioclin, Brazil), with absorbance reading performed on a spectrophotometer (INNO).

To quantify collagen accumulation as a measure of liver fibrosis, livers underwent routine histological processing for paraffin inclusion, and 5 µm thick deparaffinized sections were stained with Sirius Red (0.1% Direct Red 80 solution, Sigma-Aldrich) for 1 h, placed in a 0.1 N HCl solution for 1 min and washed with distilled water. Next, the sections were counterstained with hematoxylin for 1 min and dehydrated with increasing concentrations of alcohol, then clarified and mounted with Entellan (Merck). To quantify collagen, images of 5 fields of 3 non-serial sections per animal were obtained, totaling 15 fields per animal. The images were captured using a light microscope with a ×40 objective (Olympus BX53). Quantification of deposited collagen was performed using the segmentation tool, selecting the red-stained areas per field, and expressed in pixels per µm^2^, using Image Pro Plus 7.0 software.

### Lung analysis

Lung samples underwent routine histological processing for inclusion in paraffin and 5 µm thick sections were stained with Hematoxylin and Eosin, dehydrated with increasing concentrations of alcohol, clarified and mounted with Entellan (Merck). Lungs were evaluated under an Olympus BX53 light microscope, and quantification was performed to analyze alveolar and interstitial neutrophils and septal thickness, using a 0-4 scoring system designed to quantify the extent of lung injury histologically, based on 10 random high-power fields (magnification of 400), considering neutrophils in the alveolar space, neutrophils in the interstitial space and septal thickening, as previously described [33]. To assess lung inflammation, immunohistochemistry for CD68 and TNF-α was performed. Deparaffinized sections were immersed in hydrogen peroxide (%) for 20 min, washed in Phosphate Buffered Saline (PBS) and immersed in citrate buffer, pH 6.0 at 60 °C for 20 min. Sections were then incubated with 2.5% blocking solution (Vectastain Universal quick kit-Vector laboratories) for 20 min. Subsequently, the sections were incubated with primary antibodies anti-CD68 (Cell Signaling, E307v) and TNF-α (Santa Cruz Biotechnologies, sc-52746), diluted in PBS/BSA 1% overnight, followed by incubation with biotinylated secondary antibody, streptavidin-peroxidase and finally revealed with DAB (3,3′-diaminobenzidine tetrahydrochloride), under observation in a light microscope. Cell nuclei were stained with hematoxylin, and slides were dehydrated, clarified and mounted with Entellan (Merck). Finally, images were captured using the Olympus BX53 light microscope. To quantify CD68 and TNF-α staining, images of 10 fields of 2 non-serial sections per animal were obtained. The images were captured using a light microscope with a ×40 objective (Olympus BX53). Quantification was performed using the segmentation tool, selecting the brown-stained areas per field and expressed in pixels per µm^2^, using Image Pro Plus 7.0 software.

### Statistical analysis

Statistical data were presented in the results plotted as boxes with medians and whiskers from the minimum to the lower quartile, and from the upper quartile to the maximum, along with mean ± standard deviation of the mean (SD) of 8 animals per experimental group. The data were statistically analyzed using a one-way ANOVA (analysis of variance) test, followed by the chosen post-test. The Shapiro-Wilk test of normality was performed for all data as well as possible outliers, excluded using the ROUT test (Q = 1%). The tests and post-tests were selected according to the result obtained in the normality test regarding the nature of data distribution. Individual data points are shown for all animals. Statistical analysis was performed using the GraphPad Prism 8 software. The value of p < 0.05 was used to consider that the differences between groups were statistically significant.

## Results

### ASC phenotyping

**FIGURE 1:**
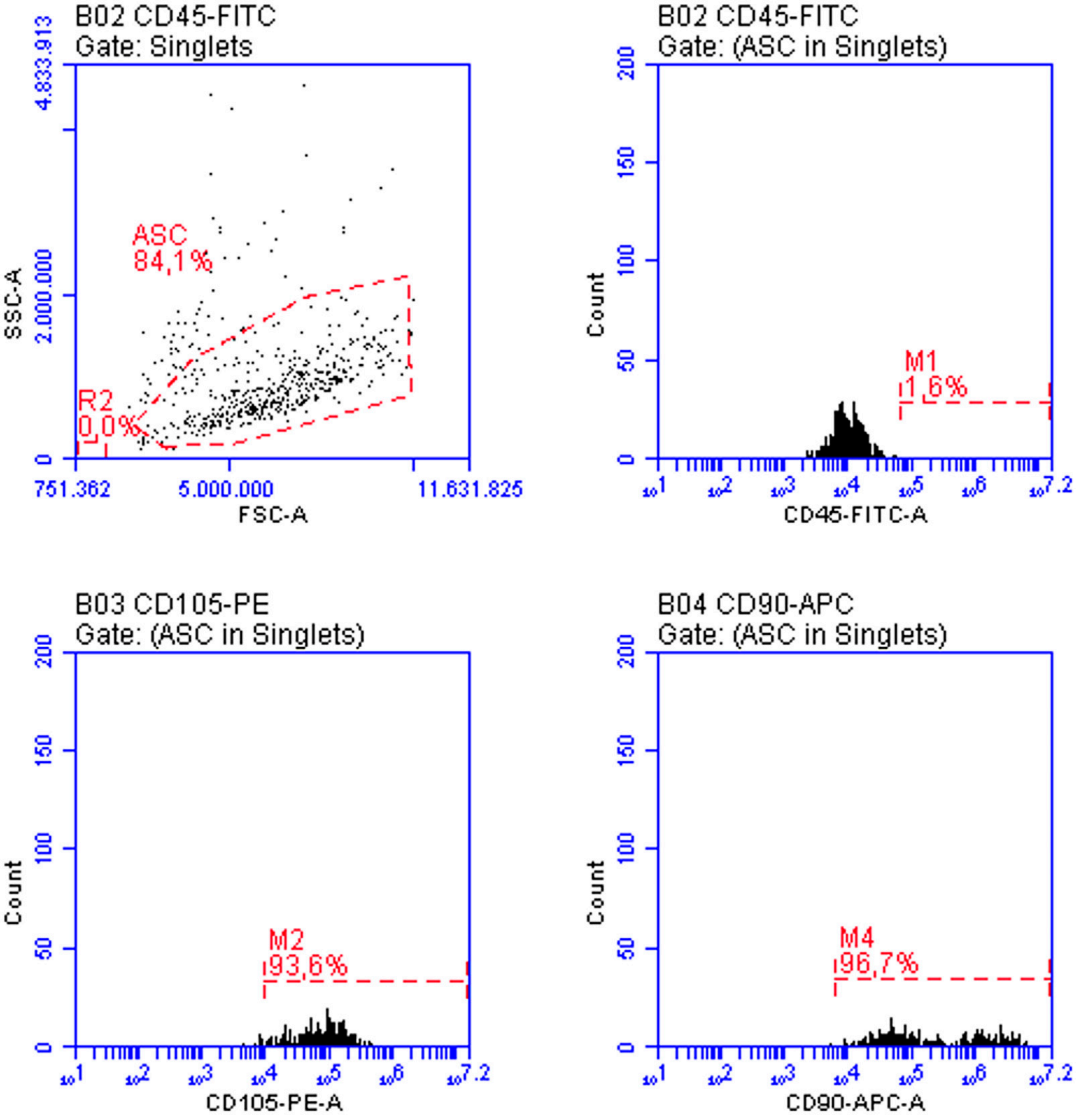
ASC characterization by flow cytometry in the third passage: more than 90% of the cells expressed varying levels of CD105 and higher levels of CD90, while CD45 expression was absent in more than 98% of the cells. These features align with the expected phenotype for these cultures.

The isolated cells were characterized in the 3^rd^ passage by flow cytometry, according to the phenotypic criteria established for murine adipose-derived mesenchymal stem cells (mASCs). Following stringent exclusion of debris and subcellular events based on scatter parameters (FSC-A vs. SSC-A) and selection of singlets (FSC-A vs. FSC-H), a morphologically homogeneous population comprising 84.1% of events was identified (Figure 1). This population was predominantly negative for the hematopoietic marker CD45 (1.6%) and exhibited high expression of the mesenchymal markers CD105 (93.6%) and CD90 (96.7%), consistent with the expected mASC phenotype. These data confirm the mesenchymal identity of the isolated cells and validate the phenotypic purity of the culture for downstream applications.

### Analysis of the ASC secretome

**FIGURE 2:**
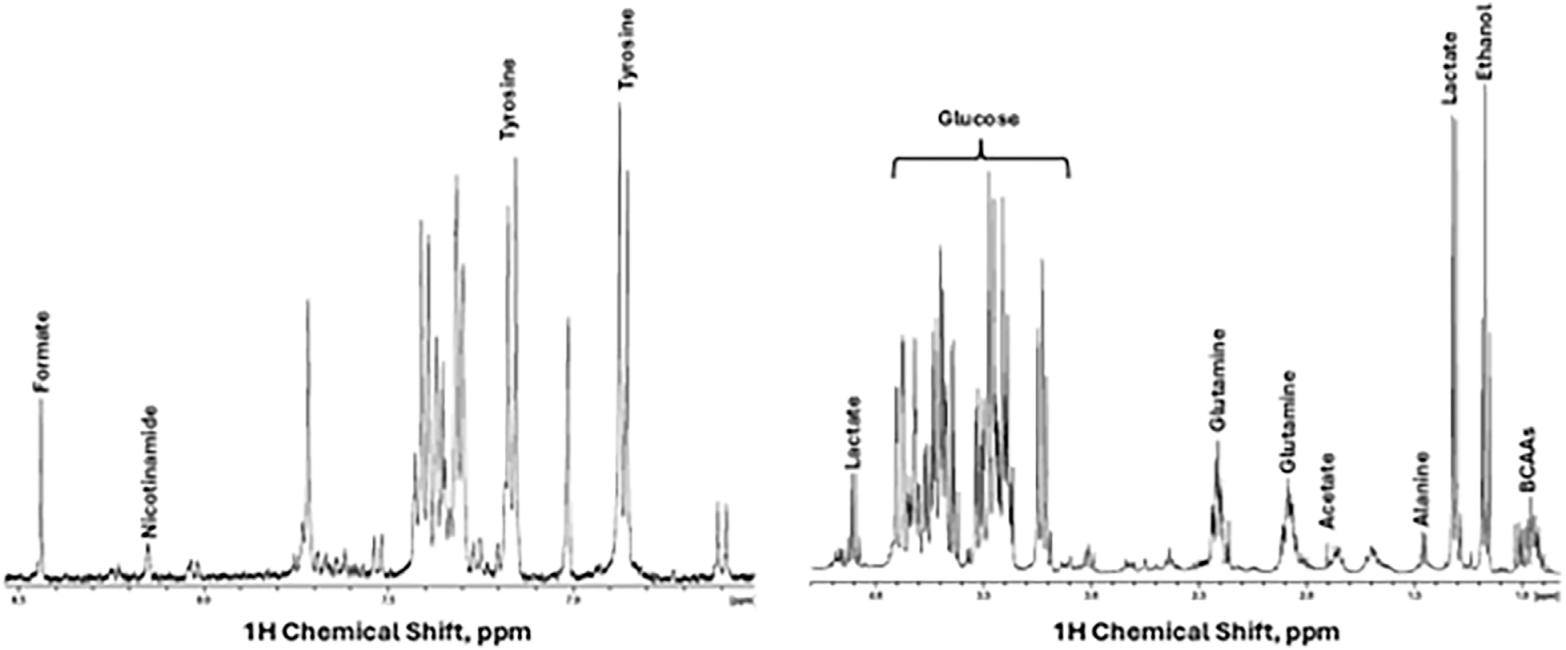
Secretome characterization. ^1^H NMR spectra display some metabolites assigned in the secretome, in the spectra aliphatic (right) and aromatic region (left).

Based on the cytokine analysis by CBA, the results indicate that ASC secretome presents varying concentrations of each analyzed cytokine, and IL-6 showed the higher concentrations, while TNF-α had the lowest levels (Table 1). The metabolomic profiling of the ASC secretome after 48 h of culture in serum-free conditions revealed a complex array of bioactive metabolites (Figure 2). Analysis by NMR spectroscopy identified key components of glycolytic metabolism, including lactic acid and pyruvic acid, consistent with the well-described preference of ASC for aerobic glycolysis. Additionally, the presence of D-glucose, maltose, rhamnose, and D-xylose suggests both residual sugar content and potential metabolic interconversions in the extracellular milieu. A broad spectrum of amino acids—such as alanine, glycine, valine, isoleucine, leucine, tyrosine, arginine, and glutamine—was detected, reflecting active protein turnover and paracrine signaling capacity. The identification of metabolites such as homoarginine, agmatine, and L-canavanine further supports the well-known role for these cells in immunomodulation. Together, these findings indicate that even under serum deprivation, ASCs sustain a metabolically active and secretory phenotype, capable of releasing a diverse set of signaling molecules relevant to tissue repair and immunoregulation.

**TABLE 1:** Analysis of cytokines in the ASC secretome using CBA kit.

Cytokines	IL-10	IL-17A	TNF-α	IFN-γ	IL-6	IL-4	IL-2
pg/mL	2.58	4.19	0.92	2.86	30.62	3.86	3.72

Interleukins 10, 17A, 6, 4 and 2, TNF-α and IFN-γ were present at varying concentrations.

### Liver analysis

**FIGURE 3:**
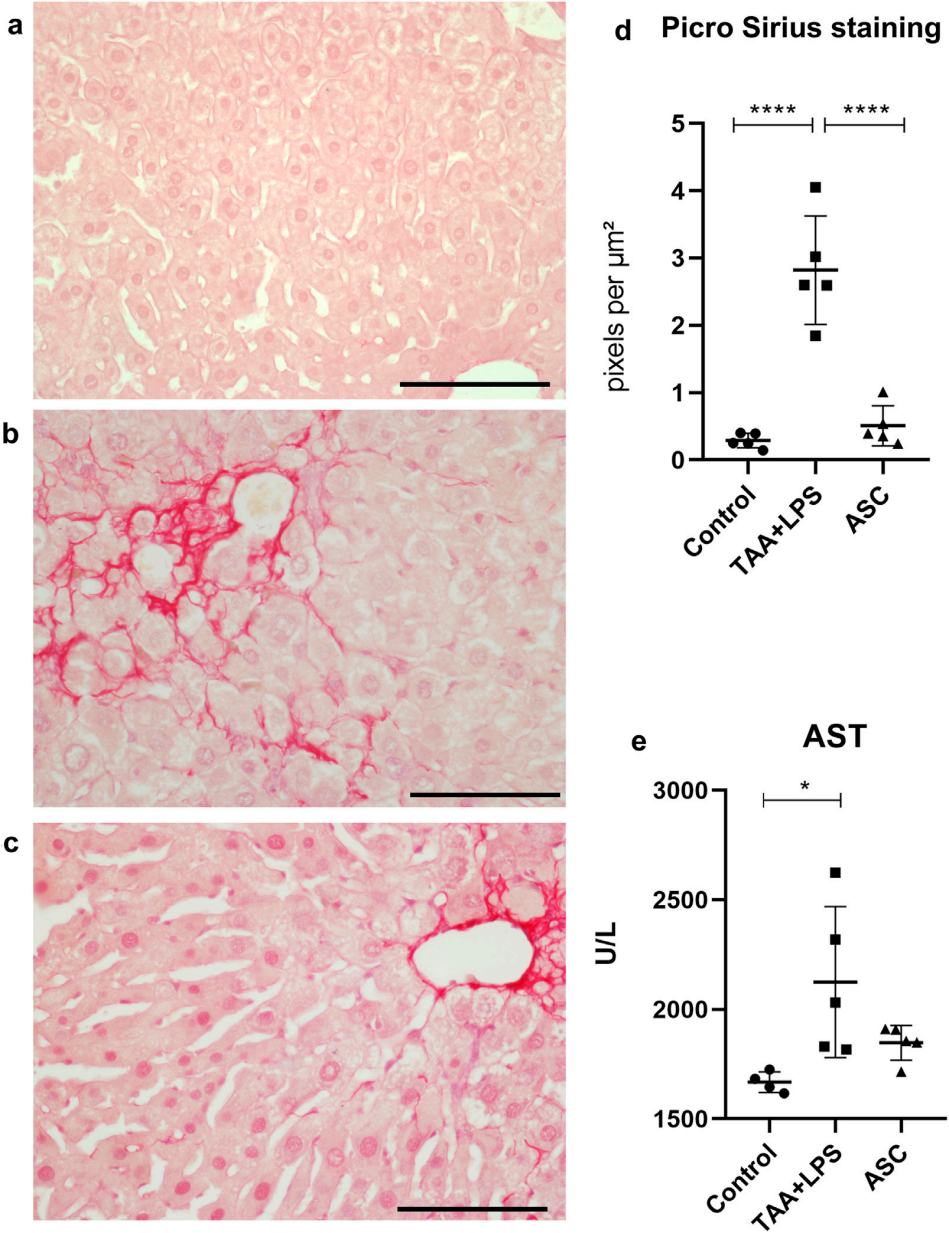
Picro Sirius was used to highlight collagen fibers in liver samples. **(a)** Control Group, with collagen restricted to portal spaces and vessels. **(b)** TAA+LPS group presented an increase in collagen fibers in red, demonstrating prominent fibrosis in the portal spaces and diffuse fibrosis in perisinusoidal areas. **(c)** ASC group, where there is a smaller amount of red-stained fibers. Bar size = 100 µm. ×40 objective. ×400 magnification. **(d)** Quantification of the stained area by densitometry: Control group (0.2884 ± 0.1087); TAA+LPS (2,822 ± 0.8072) and ASC (0.5086 ± 0.2993). **(e)** AST plasma levels: Control group (1668 ± 46.91), TAA+LPS (2124 ± 345.4) and ASC (1848 ± 79.37). Data are presented as mean ± SD; each point represents an individual animal. Statistical analysis performed with one-way ANOVA followed by Tukey’s post-test, ****P < 0.0001, *P < 0.05.

The Picrosirius (Sirius Red) staining technique was used to evaluate fibrosis extension. The presence of collagen fiber deposits was found in a basal quantity in the livers of the control group (Figure 3a). However, the TAA+LPS group showed the presence of fibrous septa and excessive collagen deposition compared to the control group, indicating the establishment of liver fibrosis in animals subjected to TAA induction. Collagen fibers were observed spreading from portal areas and around hepatocyte plates, accumulating in the perisinusoidal space (Figure 3b). Statistical analysis revealed a significant reduction in collagen deposition in the ASC group, approaching the baseline levels observed in the control group (Figures 3c,d). The analysis of AST, a liver enzyme, in blood plasma demonstrated a significant increase in the TAA+LPS group compared to the control, and although no increase was observed in the treatment with the supernatant during the analyzed period, ASC group levels of AST in the plasma were similar to the control animals (Figure 3e).

### Assessment of lung injury and inflammation

Using routine staining, it was possible to recognize and quantify three histological parameters of ALI using score counting: (1) thickening of the interalveolar septa; (2) the presence of interstitial leukocyte infiltration, especially of neutrophils and lymphocytes, both within the interalveolar septa and in the proximity of vessels and bronchioles; and (3) the presence of leukocytes, notably neutrophils, within bronchioles, alveolar ducts and alveoli. Regarding the formation of hyaline membrane and fibrin deposits, these characteristics were not identified during our analyses, at 24h, 48h and 4 days after the injury.

**FIGURE 4:**
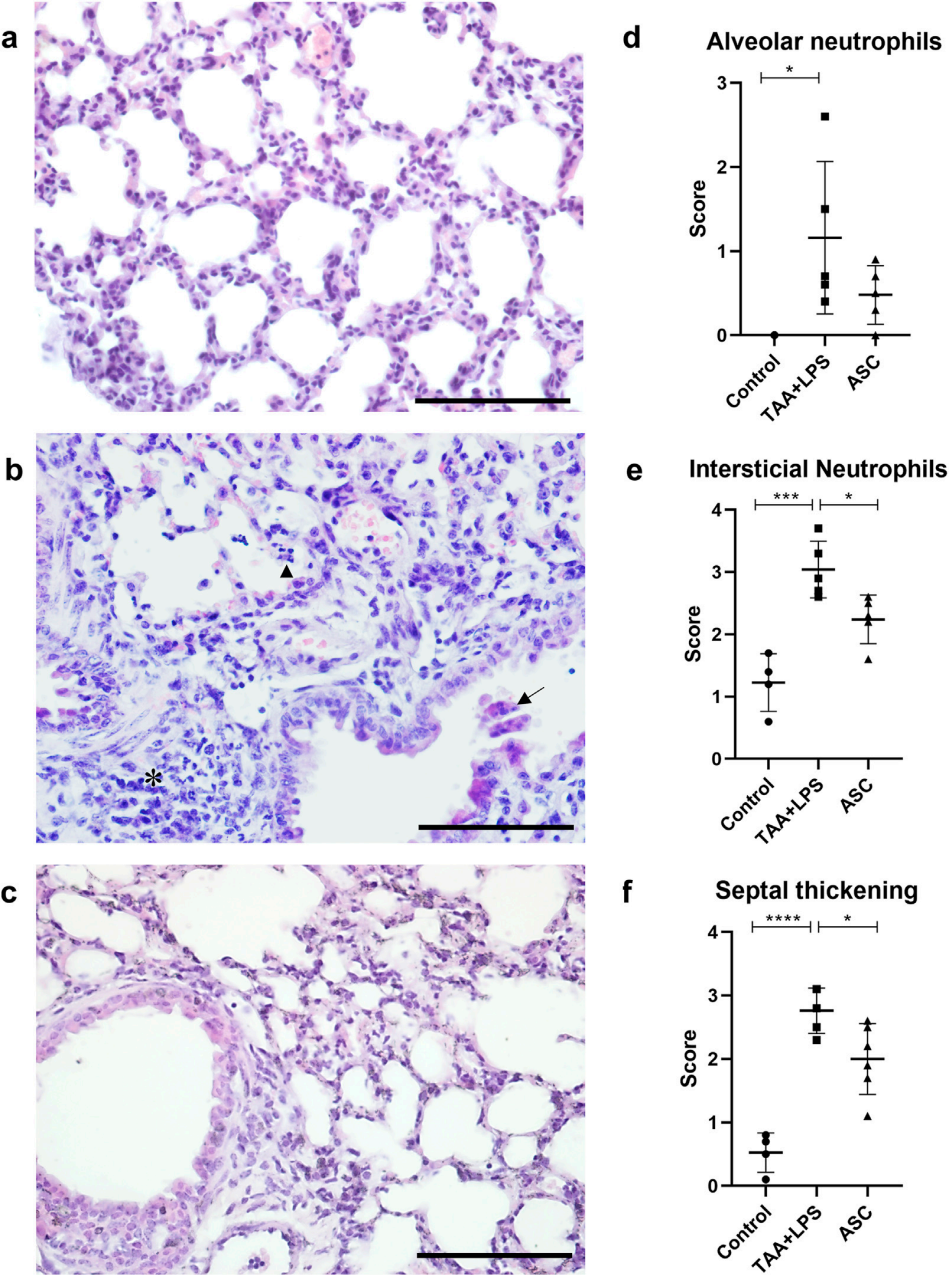
Histological analysis of the lung. **(a)** Control Group, with normal cytoarchitecture of the alveolar septa. **(b)** TAA+LPS group, diffuse thickening of the interalveolar septa, inflammatory infiltration (*), leukocytes in the bronchiolar space (arrow) and neutrophils in the alveolar space (arrowhead). **(c)** ASC group, where bronchioles without congestion were observed, and a significant reduction in the thickness of the interalveolar septa and the number of inflammatory cells. Bar size = 100 µm. ×40 objective. ×400 magnification. **(d–f)**, results of counting by scores of lung parameters, respectively: alveolar neutrophils (Control, 0.000 ± 0.000, TAA+LPS, 1,160 ± 0.9072 and ASC, 0.4800 ± 0.3493), interstitial neutrophils (Control, 1,225 ± 0.4646, TAA+LPS, 3,040 ± 0.4561 and ASC, 2,240 ± 0.3912) and thickening of the interalveolar septum (Control, 0.5250 ± 0.3096, TAA+LPS, 2,760 ± 0.3578 and ASC, 2,000 ± 0.5586). Data are presented as mean ± SD; each point represents an individual animal. Statistical analysis performed with one-way ANOVA followed by Tukey’s post-test, ****P < 0.0001, ***P < 0.001, and *P < 0.05.

We observed that after injury with LPS, there is an abrupt and significant increase in the three parameters evaluated (Figure 4), and that treatment with ASC secretome was able to significantly reduce interstitial neutrophils and thickening of the interalveolar septum. The photomicrographs demonstrate the remodeling of the lung parenchyma, with recovery of the alveolar morphology lost after the injury, and a reduced amount of inflammatory infiltration.

**FIGURE 5:**
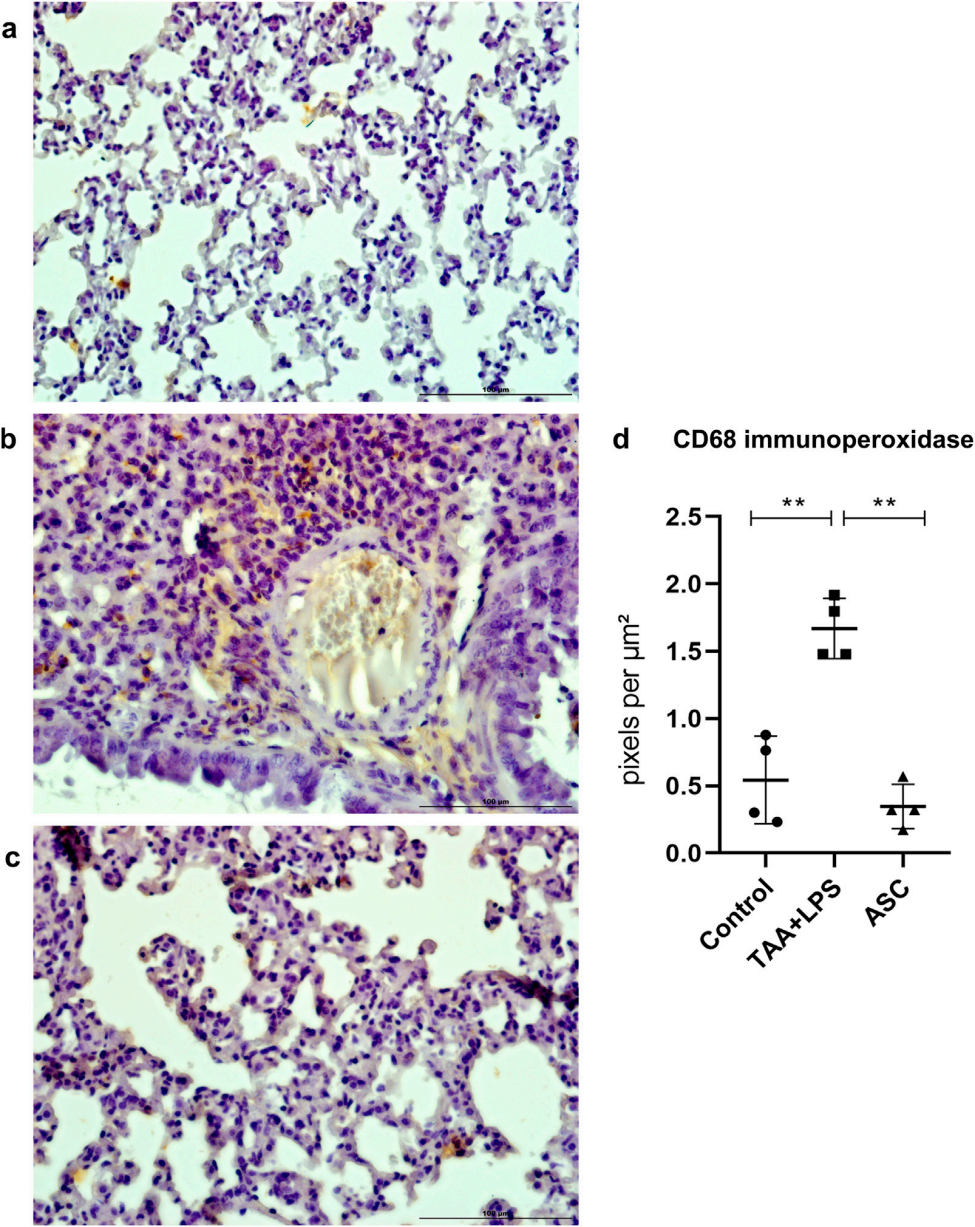
Immunoperoxidase to detect CD68 protein expression in the lung. **(a)** Control Group, with normal cytoarchitecture of the alveolar septa and basal levels of CD68 expression. **(b)** TAA+LPS group, with greater staining for CD68, especially in regions of inflammatory infiltration. **(c)** ASC group, reduction of inflammatory cells marked with CD68. Bar size = 100 µm. ×40 objective. ×400 magnification. **(d)** CD68 labeling quantification showed that ASC secretome is effective in reducing tissue macrophages. Control (0.5436 ± 0.3253), TAA+LPS (1,731 ± 0.2267), and ASC (0.3556 ± 0.2002). Data are presented as mean ± SD; each point represents an individual animal. Statistical analysis performed with one-way ANOVA followed by Tukey’s post-test, **P < 0.01.

The results of immunohistochemistry for CD68, a lung macrophage marker, confirmed the expected staining findings related to tissue inflammation. There was a significant increase in expression after injury with LPS (Figure 5), and a reduction in labeling after treatment with ASC secretome. Furthermore, in the injured group, we observed a large number of macrophages clustered in areas of inflammation, while in control and treated animals, macrophages were found in the usual location, associated with the alveolar space.

**FIGURE 6:**
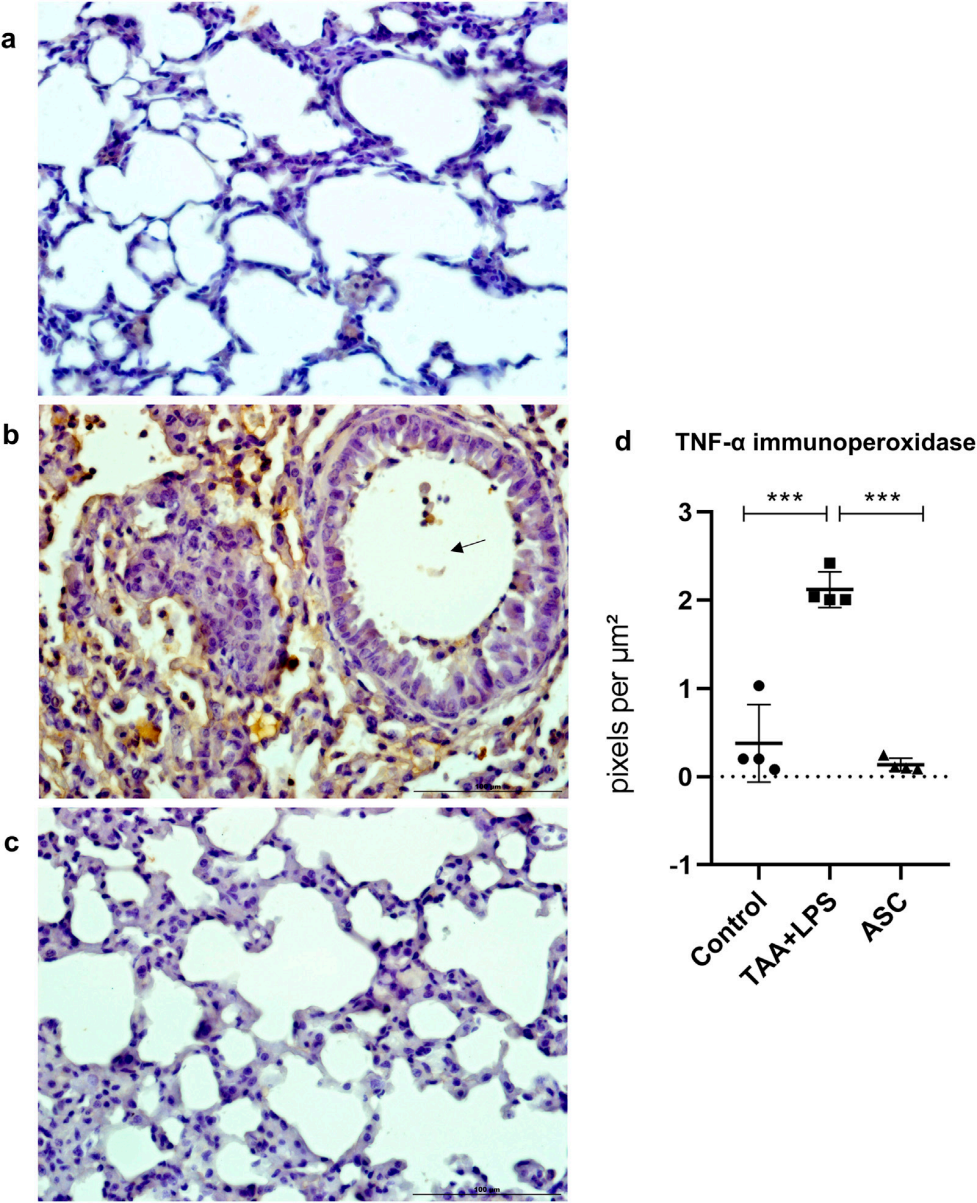
Immunoperoxidase to detect TNF-α protein expression in the lung. **(a)** Control Group, with normal cytoarchitecture of the alveolar septa and basal levels of TNF-α expression. **(b)** TAA+LPS group, with greater staining for TNF-α, diffusely around bronchioles and thickened interalveolar septa. Marking is also observed in leukocytes inside a bronchiole (arrow). **(c)** ASC group, with lower expression of TNF-α and preserved alveolar cytoarchitecture. Bar size = 100 µm. ×40 objective. ×400 magnification. **(d)** TNF-α labeling quantification showed that ASC secretome is effective in reducing inflammation sites in the lung parenchyma. Control (0.4378 ± 0.5174), TAA+LPS (2.157 ± 0.2296), and ASC (0.1367 ± 0.07313). Data are presented as mean ± SD; each point represents an individual animal. Statistical analysis performed with one-way ANOVA followed by Tukey’s post-test, ***P < 0.001.

TNF-α is an inflammatory cytokine of relevance as a predictor of loss of lung function and injury severity. Similar to CD68 labeling, marked and diffuse expression occurred after LPS injury, which was significantly reduced in animals treated with the ASC secretome (Figure 6).

### Plasma cytokine analysis

**FIGURE 7:**
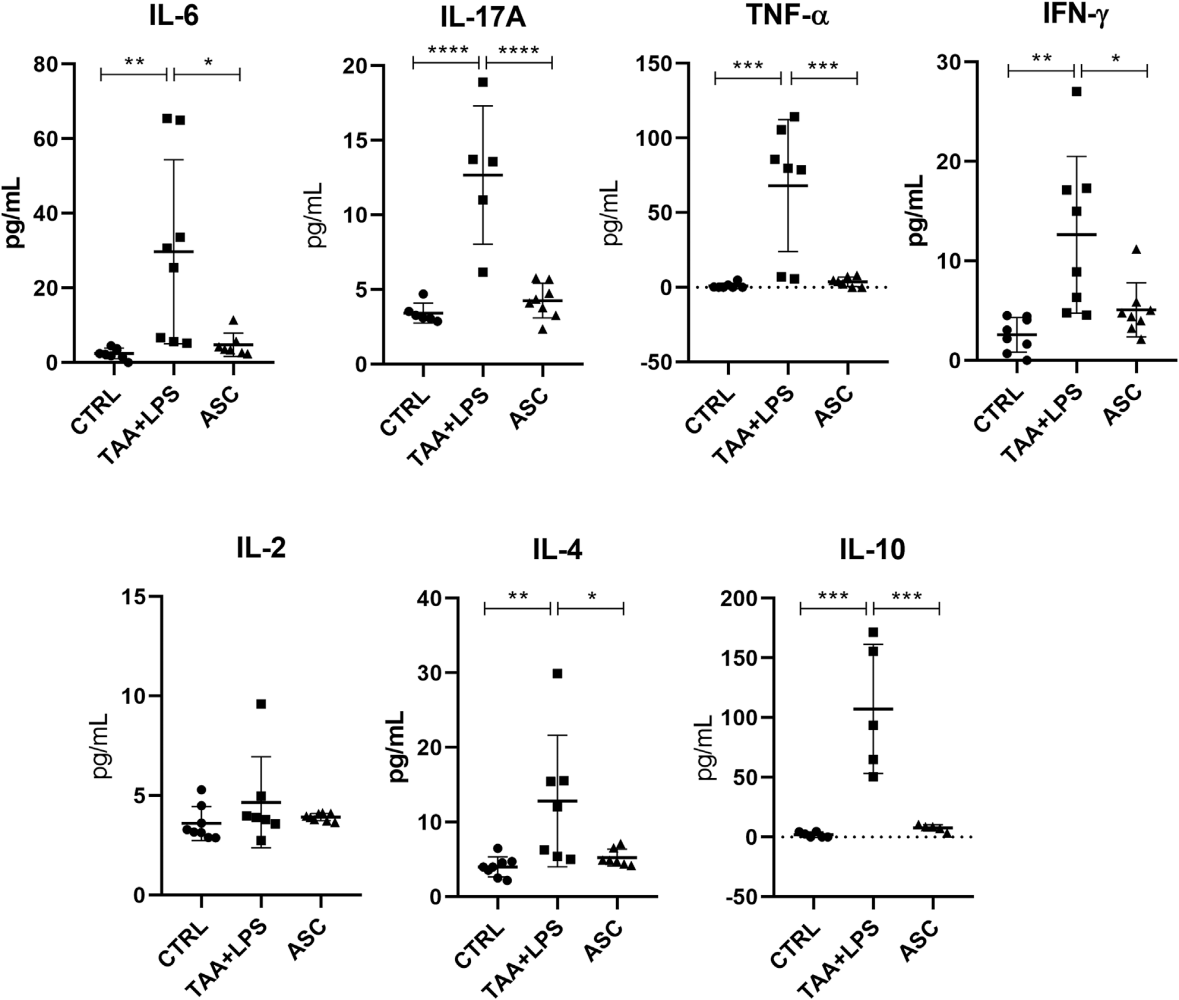
IL-6, IL-17A, TNF-α, IFN-γ, IL-2, IL-4, and IL-10 plasma levels were analyzed in a flow cytometer using the CBA kit (BD Biosciences) for Th1, Th2 and Th17 responses, and are expressed as a concentration of pg/mL. Data are presented as mean ± SD; each point represents an individual animal. Statistical analysis performed with one-way ANOVA followed by Tukey’s post-test, ****P < 0.0001, ***P < 0.001, **P < 0.01, and *P < 0.05.

There was a significant increase in plasma levels of the cytokines IL-10, IL-17A, IFN-γ, TNF-α, IL-6, IL-4 in the TAA+LPS group when compared to the control group, and a marked reduction of all these cytokines was observed in the ASC group compared to the TAA+LPS group (Figure 7). As for IL-2, statistical tests did not reveal significant differences between groups. All results obtained in animals treated with ASC secretome demonstrated a return of cytokine concentrations to levels close to control animals, indicating a systemic immunomodulatory effect from ASCs secretome.

### Liver cytokine analysis

**FIGURE 8:**
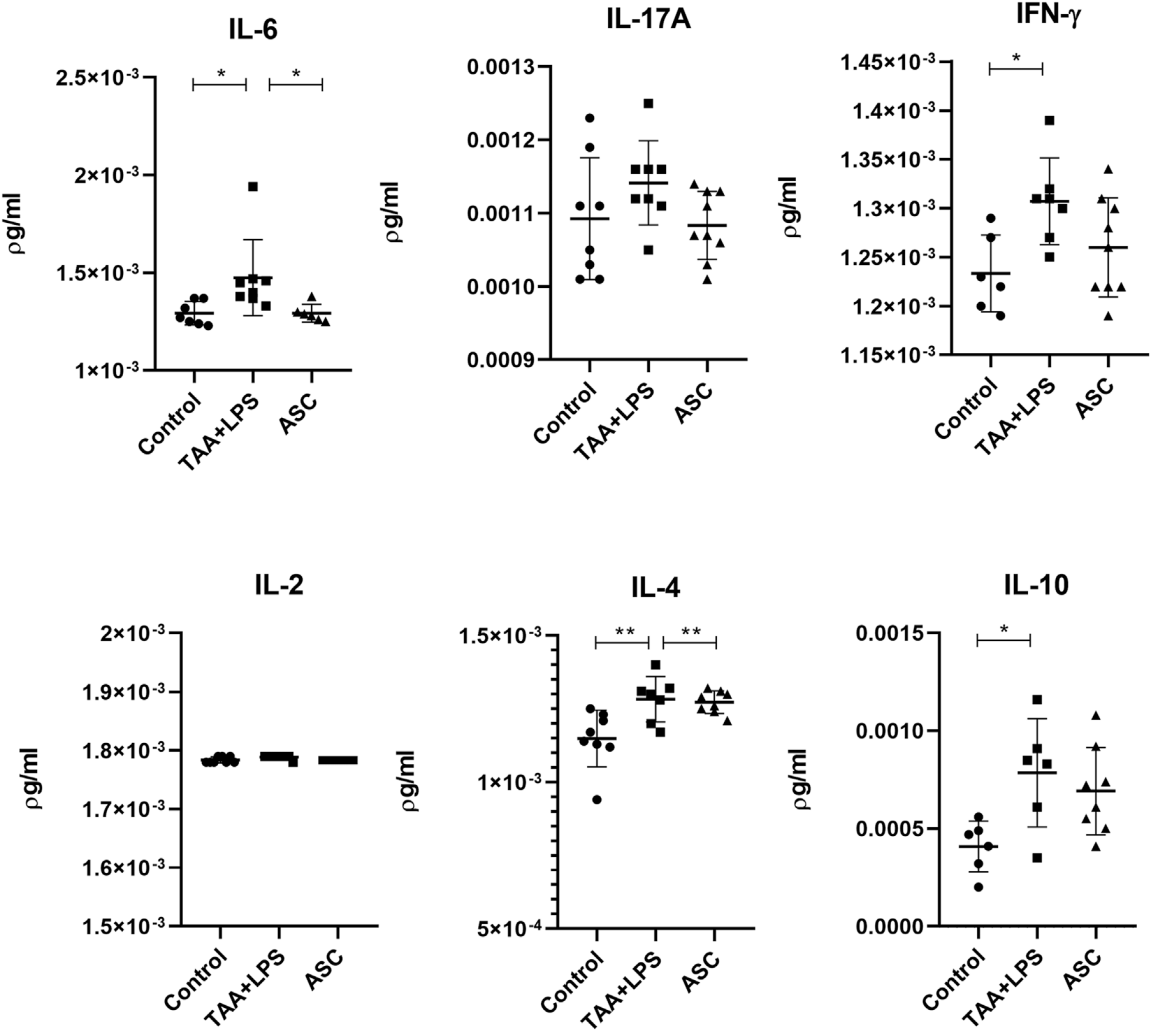
Levels of IL-6, IL-17A, IFN-γ, IL-2, IL-4, and IL-10 in liver lysates were quantified by flow cytometry using the CBA kit (BD Biosciences) to assess Th1, Th2, and Th17 responses, and are expressed as pg/mL. Data are presented as mean ± SD; each point represents an individual animal. Statistical analysis was performed using one-way ANOVA followed by Holm–Sidak’s post-test. **P < 0.01, *P < 0.05.

TAA administration was associated with an increase in IFN-γ, IL-4, IL-6 and IL-10 levels in liver tissues of the TAA+LPS group. However, ASC secretome correlated with decreased levels of these cytokines, especially IL-6, showing an improvement towards a decreased inflammatory activity (Figure 8).

### Lung cytokine analysis

**FIGURE 9:**
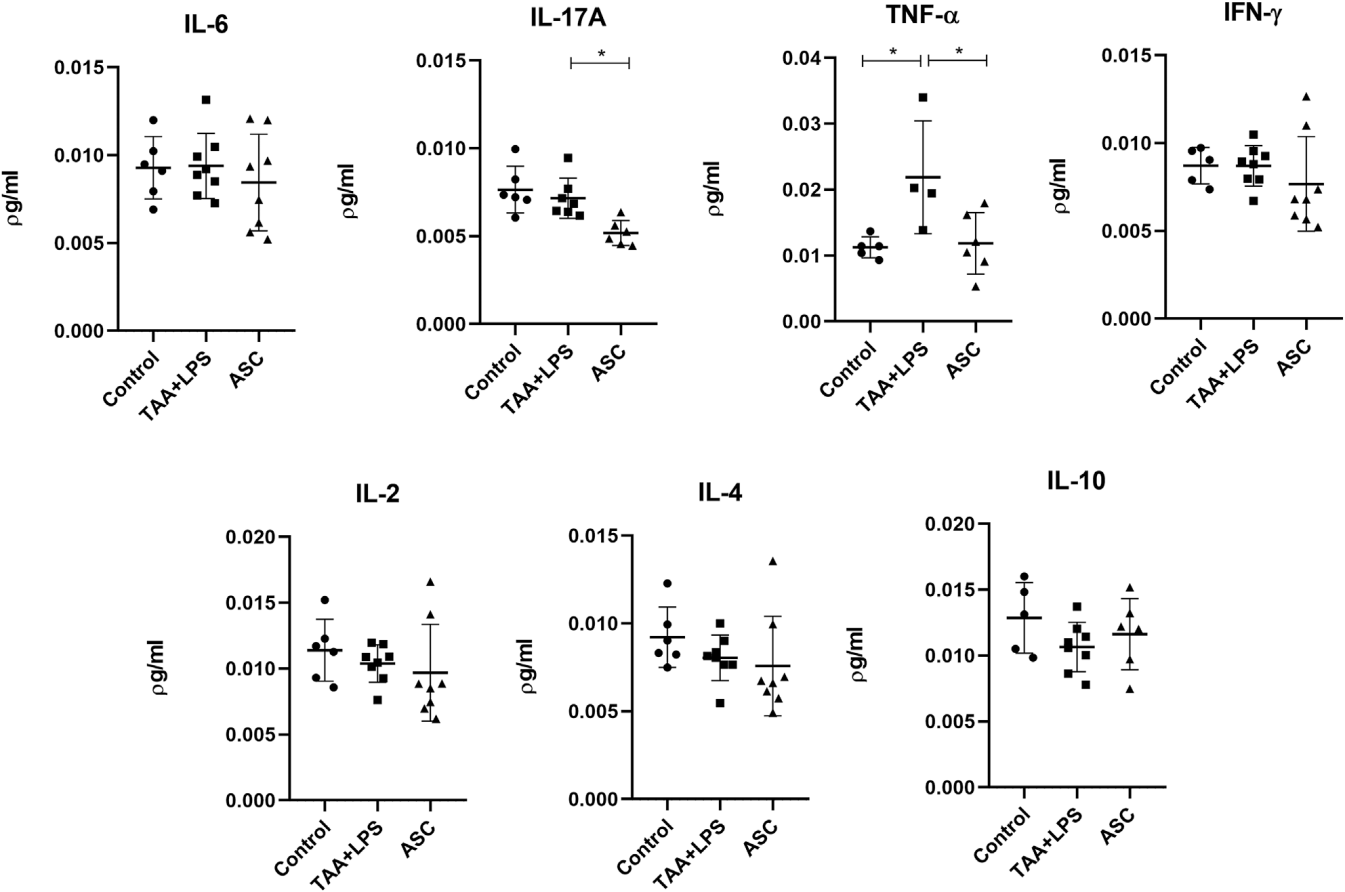
Levels of IL-6, IL-17A, TNF-α, IFN-γ, IL-2, IL-4, and IL-10 in lung lysates were analyzed in a flow cytometer using the CBA kit (BD Biosciences) for Th1, Th2 and Th17 responses, and are expressed as a concentration of pg/mL. Data are presented as mean ± SD; each point represents an individual animal. Statistical analysis performed with one-way ANOVA followed by Holm–Sidak’s post-test, **P < 0.01, and *P < 0.05.

Results showed that ASC treatment correlated with significantly decreased TNF-α and IL-17A levels in the lung, 7 days after acute LPS-induced inflammation and 6-weeks chronic TAA exposure (Figure 9).

## Discussion

In this work, ASC secretome effects in a model of concomitant liver fibrosis and ALI were assessed to evaluate its therapeutic potential in chronic-acute disease. Although there is much data confirming that ASC paracrine modulation helps ameliorate both chronic and acute disorders, for the first time we demonstrated that this is also possible in a murine model of a CLD-ALI scenario, where both injured liver and lung were benefited for ASC secretome administration, as evidenced by a significant decrease in liver fibrosis and in inflammatory activity in the lung as well as in the systemic circulation. It is noteworthy that liver fibrosis by TAA and ALI by LPS have completely unique features, having in common a prominent inflammatory background, but in each case, different inflammatory cells and molecules are involved. Nevertheless, there is significant interchange between the liver and lung circulatory axis [34, 35], and this feature must also be considered when these organs are affected.

CLD is a condition characterized by continuous and sustained inflammation leading to fibrosis and structural liver changes. Liver fibrosis, triggered by various factors, poses a significant health challenge, as it can progress to cirrhosis and hepatocellular carcinoma. This process involves the activation of Kupffer cells and hepatic stellate cells, and abnormal extracellular matrix deposition [1, 22, 36]. Different chemical compounds like CCl_4_, dimethylnitrosamine and TAA are used to induce liver fibrosis in animal models. Each model has unique characteristics and provides valuable insights for effective therapeutic strategies [32].

ALI, whether associated or not with acute respiratory distress syndrome (ARDS), has serious consequences and high mortality rates, and inflammation as well as oxidative stress play a significant role [37, 38]. The liver-lung interplay is little explored, and bacterial endotoxins such as LPS - commonly found in the gut and a hallmark of severe bacterial infection and sepsis - are largely absorbed by the mesenteric veins, which carry the toxins to the liver, and further to systemic circulation, from which they can lead to severe lung damage [34, 39–41]. Therefore, LPS is widely used to study acute lung injuries, as it may mimic the histopathological features of human lung injury induced by respiratory pathogens. LPS can also potentially lead to hepatic compromise in cirrhotic individuals, increasing the severity and mortality rate of the disease [42–45].

MSC have been the focus of intensive research for clinical applications aimed at tissue regeneration, metabolic and musculoskeletal disorders, acute and chronic inflammatory and autoimmune diseases, with prospects for innovative discoveries. MSC secretome consists of diverse bioactive molecules, including small and large extracellular vesicles, cytokines, and growth factors [18, 20, 46]. These substances have the capability to influence processes such as tissue regeneration, immune response, angiogenesis and inflammation. They can modulate the inflammatory response and reduce fibrogenesis, affecting both the local and systemic environment when released by MSC. There is increasing interest in defining the content of exosomes in MSC secretome and in developing strategies to escalate its production, for use as a potential therapeutic treatment, with the advantage of being an acellular product, thus presenting greater safety for clinical use [27, 28, 47, 48].

The major concerns regarding cell therapies are defining the complex interactions between donor and recipient’s cells and establishing the complete composition of the cell solution obtained in each therapeutic protocol. When secretome is applied, these concerns are more easily assessed, because the complexity is reduced from live cells to simply secreted molecules and vesicles, in a solution that is suitable for proteomic screenings [46, 48]. Bearing this in mind, this work aimed at the potential benefits of MSC secretome in multiple organs and pathological conditions after peritoneal administration and absorption, proving that this is an easy and effective way of delivering MSC derivatives, independently of the target organ. An interesting aspect in this choice is that MSC molecules and exosomes are easily absorbed through the peritoneal membrane, and particularly exosomes possess surface receptors that help deliver them to injured and inflamed sites via circulation, after absorption [47]. Alternatively, if ASCs hole fraction was to be used, the best access pathway would be by vascular administration, implying a greater risk of thromboembolic events [46].

The effectiveness of MSC depends on several factors, and this is a sort of heterogeneous and complex population in terms of phenotypic characterization (such as a non-exclusive CD105, CD90, CD73 expression and exclusion of hematopoietic markers) and functional roles (the multipotent capacity to differentiate through fibrogenic, adipogenic, chondrogenic, or osteogenic processes under certain stimuli). Today, MSC are considered the progeny of *bona fide* mesenchymal stem cell precursors that are present in some tissues at an extremely low rate [19]. MSC therapeutic capacity is influenced by different aspects of the organism, namely the original tecidual source, age, general metabolic state and preexisting chronic conditions [46, 47]. There is evidence that MSC exert their beneficial roles through the paracrine way. This effect is observed in both animal and clinical studies, for a vast myriad of diseases of diverse etiologies [18, 20, 46, 47, 49]. Our group has published evidence on MSC therapeutic effect on liver fibrosis previously [25, 50], as well as many worldwide groups in different models and experimental designs, including acute liver injury [22, 24, 26]. This approach was proven true for different models of lung injury likewise [29, 51–55], however, for the first time we observed in a murine preclinical model that the ASCs secretome can exert its therapeutic effects simultaneously and systemically in both liver and lung disease, which points to a promising and safe strategy when conventional pharmacological therapy is complicated by multiorgan injury.

MSC secretome stimulates fibrosis regression through multiple pathways, leading to immunomodulation towards M2 phenotype in tecidual macrophages, which is a first kick towards fibrogenic cell deactivation and apoptosis, favoring tissue remodeling in a beneficial way. The common cytokine repertoire in this scenario may include unexpectedly high levels of interleukin IL-6 and low levels of IL-10, which stimulates hepatocyte proliferation and hepatic remodeling in CLD [22, 24, 26]. In the present study, we aimed to answer whether ASC could still be effective for liver regeneration in a severe lung injury situation, and histological as well as plasma analysis showed that the secretome maintained its effect in the liver during ALI, although we believe that liver regeneration appeared slower or in a lesser extent because we expected a more pronounced reduction in AST plasmatic levels in the ASC treated group. This underscores the need to assess durability and dosing frequency in future studies, as repeated administration has been shown to prolong therapeutic effects. In this respect, although repeated dosing has been shown to prolong therapeutic benefits in chronic injury models [27], our study focused on demonstrating that a minimal secretome dose was sufficient to achieve systemic and hepatic effects, and future work should address whether additional administrations could further enhance recovery. Several chronic conditions in the liver can lead to loss of pulmonary function even in the absence of a pathogenic infectious agent, such as hepatopulmonary syndrome observed in cases of severe cholestasis and/or portal hypertension, in NAFLD, decompensated cirrhosis and in chronic infections with hepatitis viruses [56–60]. This observation enhances the multiuse advantages of using MSC secretome in systemic delivery for CLD patients. In our study, LPS induction resulted in a massive injury to the lung parenchyma, with widespread disorganization of interalveolar septa and other inflammatory features such as a marked increase in CD68 and TNF-α levels, as observed by other authors [42–44]. All these features were obviously attenuated after ASC secretome treatment, disregarding the preexisting CLD condition, showing that the reduction in lung macrophages is associated with TNF-α decrease, in an overall decline of the inflammatory activity inflicted by LPS. This was even more encouraging by the observation that all plasmatic cytokines that were quite imbalanced in the TAA+LPS group returned to normal basal levels after the secretome therapy. In fact, the well-known cytokine storm is a common cause of severe and fatal lung and multiorgan injury, including acute liver failure [4, 13, 61], and LPS administration simulated a similar scenario detectable in the altered cytokine plasmatic levels in this study.

Of the seven cytokines related to Th1, Th2 and Th17 responses measured in the present work, only cytokine IL-2 was not affected in the plasma, nor by our experimental disease model, nor by the secretome treatment. This is because this protein is more related to lymphocyte activation in pathogenic infections, and although LPS is of bacterial origin, it does not lead to a complete immune response [44, 62]. It is noteworthy that the main cytokines elevated in our injury model are IL-10, IL-17A and TNF-α, what denotes the diverse nature of the inflammatory response in this case, since there are two different diseases involved, both in major organs that impact directly in systemic parameters, and of diverse cause (chronic and acute). Cytokines IL-6 and IL-10, although thought of generally having inflammatory and anti-inflammatory properties respectively, play very diverse roles in the local and systemic regulation of the immune response and bone marrow hematopoiesis [63–65]. These cytokines can promote both pro-inflammatory and anti-inflammatory responses according to the local microenvironment status and cell interactions influencing their production [64, 65]. The normalization of all plasmatic cytokines affected by chronic TAA and acute LPS administration to levels similar to the control group in the ASC group points to a significant immunomodulation capacity of the ASC secretome in regulation of the systemic immune response, confirming that this therapy which may be benefic even in respiratory infectious diseases [66].

Further exploration of systemic immunomodulation remains essential to strengthen the translational relevance of our findings. While the observed normalization of circulating cytokines indicates broad immune rebalancing, more detailed analyses—such as longitudinal cytokine kinetics, flow cytometry of immune cell subsets, and functional assays of T cells, macrophages, and NK cells—would help delineate the mechanisms by which MSC-derived products exert their effects. Such approaches could clarify whether the modulation is transient or sustained, specific to certain pathways, or generalized across the immune network. Future studies integrating multi-omics profiling and temporal resolution will be critical to validate systemic immunomodulation as a core mechanism underlying therapeutic benefit [20].

Our metabolomic profiling of ASC secretome further supports a mechanistic link with the immunomodulatory effects observed. The presence of agmatine, reported to attenuate pro-inflammatory signaling and nitric oxide synthase activity [67], may have a role with the reduction in IL-6 and TNF-α detected in plasma and tissues. Likewise, homoarginine, which has been associated with endothelial protection and antifibrotic processes [68], may contribute to the tissue-repair context observed in the liver and lung. Beyond these specific metabolites, the abundance of amino acids and arginine-derived compounds suggests engagement of pathways linked to redox balance and immune regulation, which aligns with the systemic normalization of cytokines after treatment. While correlative, these findings reinforce the hypothesis that secretome bioactivity involves a coordinated metabolic–immune interface that merits further targeted validation.

Regarding liver and lung tissue levels of the measured cytokines, it is noteworthy that given the time point of analysis (7 days after injuries), early and minor local changes in the tissues were not scored. However, after ASC treatment, we observed a decrease in the main cytokines involved in inflammatory processes in each organ and model, as for TNF-α in acute lung injury (confirming immunoperoxidase results), and IL-6 in chronic liver fibrosis. These data show that ASC can attenuate different inflammatory processes in diverse organ systems, revealing a multifaceted effect which proved to be efficient in reverting injuries in the different pathological models applied in this study.

The differential cytokine responses observed in liver and lung allow us to hypothesize potential cellular and molecular pathways involved. The reduction of hepatic IL-6 may reflect modulation of Kupffer cells and hepatic stellate cells, consistent with a dampening of STAT3-driven inflammatory signaling. In parallel, the decrease in pulmonary TNF-α suggests inhibition of NF-κB activity, a central axis in alveolar macrophage–mediated inflammation [29]. While our data do not directly establish these mechanisms, proposing such links highlights plausible immune and stromal cell populations as targets of secretome activity and provides a framework for future mechanistic investigation.

In comparison with existing MSC-based therapies, our findings underscore key distinctions among live-cell administration, exosome-enriched products, and unfractionated secretome. Direct infusion of MSCs has been extensively tested and offers regenerative and immunomodulatory benefits, but concerns remain regarding engraftment efficiency, potential for ectopic differentiation, and risks such as microvascular occlusion [18]. Exosome-based strategies, in turn, provide mechanistic precision through well-defined vesicular cargo but are limited by complex and costly isolation procedures that challenge large-scale clinical translation. Unfractionated secretome integrates soluble proteins, metabolites, and extracellular vesicles, retaining broad therapeutic activity while offering advantages in manufacturing scalability and accessibility, which are particularly relevant in resource-limited health systems [69, 70]. This layered comparison highlights that secretome-based approaches may combine safety and feasibility benefits while reducing the risks associated with cell-based administration. In summary, our findings reinforce the therapeutic potential of ASC-derived secretome in a preclinical setting, suggesting it may represent a minimally processed, cell-free strategy applicable to inflammatory and fibrotic conditions. Importantly, the use of unfractionated secretome underscores its possible translational relevance in settings where advanced processing technologies for exosome or EV isolation are technically or economically prohibitive. This aligns with the urgent need for scalable and accessible regenerative interventions in real-world clinical systems [69].

## Conclusion

In conclusion, this study provides evidence that ASC secretome therapy may be a helpful tool in the treatment of liver fibrosis and ALI. Its potential to modulate the inflammatory response both systemically and locally, promote tissue regeneration, and attenuate fibrosis makes it a promising approach for these conditions, although the mechanistic effects and protocol improvement demand further investigations.

Despite promising results, there are challenges to overcome before MSC secretome therapy is widely adopted in clinical practice. Additional studies are needed to define secretome composition and to understand the mechanisms of action involved in its regenerative and immunomodulating responses, before conducting controlled clinical trials to assess efficacy and safety in human patients.

## Data Availability

The original contributions presented in the study are included in the article/supplementary material, further inquiries can be directed to the corresponding author.

## Footnotes

1
http://www.metabogen.com.br
